# Data Extractions Using a Large Language Model (Elicit) and Human Reviewers in Randomized Controlled Trials: A Systematic Comparison

**DOI:** 10.1002/cesm.70033

**Published:** 2025-06-08

**Authors:** Joleen Bianchi, Julian Hirt, Magdalena Vogt, Janine Vetsch

**Affiliations:** ^1^ Department of Health Eastern Switzerland University of Applied Sciences St. Gallen Switzerland; ^2^ Interdisciplinary Infant Unit Eastern Switzerland Children's Hospital St. Gallen Switzerland; ^3^ Pragmatic Evidence Lab, Research Center for Clinical Neuroimmunology and Neuroscience Basel (RC2NB) University Hospital Basel University of Basel Basel Switzerland; ^4^ Institute of Health and Nursing Science, Medical Faculty Martin Luther University Halle‐Wittenberg Halle (Saale) Germany

**Keywords:** artificial intelligence, data extraction, human reviewer, randomized controlled trial, systematic review

## Abstract

**Aim:**

We aimed at comparing data extractions from randomized controlled trials by using Elicit and human reviewers.

**Background:**

Elicit is an artificial intelligence tool which may automate specific steps in conducting systematic reviews. However, the tool's performance and accuracy have not been independently assessed.

**Methods:**

For comparison, we sampled 20 randomized controlled trials of which data were extracted manually from a human reviewer. We assessed the variables study objectives, sample characteristics and size, study design, interventions, outcome measured, and intervention effects and classified the results into “more,” “equal to,” “partially equal,” and “deviating” extractions. STROBE checklist was used to report the study.

**Results:**

We analysed 20 randomized controlled trials from 11 countries. The studies covered diverse healthcare topics. Across all seven variables, Elicit extracted “more” data in 29.3% of cases, “equal” in 20.7%, “partially equal” in 45.7%, and “deviating” in 4.3%. Elicit provided “more” information for the variable study design (100%) and sample characteristics (45%). In contrast, for more nuanced variables, such as “intervention effects,” Elicit's extractions were less detailed, with 95% rated as “partially equal.”

**Conclusions:**

Elicit was capable of extracting data partly correct for our predefined variables. Variables like “intervention effect” or “intervention” may require a human reviewer to complete the data extraction. Our results suggest that verification by human reviewers is necessary to ensure that all relevant information is captured completely and correctly by Elicit.

**Implications:**

Systematic reviews are labor‐intensive. Data extraction process may be facilitated by artificial intelligence tools. Use of Elicit may require a human reviewer to double‐check the extracted data.

## 1 Introduction

Systematic reviews are considered the most reliable method for synthesizing evidence, as they adhere to a structured, rigorous, and transparent research process. Due to their thoroughness, systematic reviews have long been pivotal in shaping health policy, clinical guidelines, and primary research [1]. The time required for a full systematic review, which is often more than 2 years after the publication of a protocol, represents a significant obstacle for both author teams and decision‐makers [2]. Artificial intelligence (AI) tools have the potential to streamline the process of conducting systematic reviews, thereby reducing the time required and the number of errors [3]. In the study by Affengruber et al. [1], the use of various tools, including Plot Digitizer, ChatGPT, ExaCT, Dextr, and DAA, was evaluated for their effectiveness in accelerating data extraction from studies. Manual data extraction by two reviewers, supplemented by the Plot Digitizer, exhibited comparable agreement with the original data, with slightly higher concordance achieved using the Plot Digitizer compared to manual extraction alone. In total, 87% of manually extracted data elements aligned with ExaCT, leading to altered outcomes in meta‐analyses. ChatGPT demonstrated consistent agreement with human researchers across various parameters, including language, target disease, natural language processing model, sample size, and performance metrics, with moderate to fair agreement observed for clinical tasks and clinical implementation. User‐friendliness was evaluated for DAA and Dextr, with both tools rated as highly user‐friendly; however, DAA scored lower on feature‐based assessments, while Dextr was noted for its flexible interface [1].

## 2 Background

There is an AI research tool called “Elicit” which, in comparison to manual data extraction by trained research staff, demonstrated an accuracy that was 13%–26% higher (Elicit). However, this accuracy data were conducted and reported by Elicit and there is—to the best of our knowledge—no full independent research report on Elicit's accuracy. Elicit leverages large language models, including GPT‐3, to automate research workflows. As the accuracy data are derived from the developers of Elicit and no alternative data are available, there is a need to evaluate its data extraction capabilities against human reviewers for a more robust assessment of its performance. Therefore, the aim is to evaluate and compare the data extraction capabilities of Elicit with those of a human reviewer to assess the accuracy and completeness of the data.

### 2.1 Design

To compare the data extraction from Elicit with human extractions, we compared 20 studies of which data were extracted manually by a human reviewer from FIT‐Nursing Care versus Elicit. This procedure was undertaken to evaluate the comparative efficacy of the predefined variables (see below). FIT‐Nursing Care is a nursing knowledge platform providing study summaries. These German summaries are developed by health researchers using predefined data extraction fields in an online platform [4].

### 2.2 Methods and Materials

#### 2.2.1 Eligibility Criteria and Data Source

We considered individual and cluster randomized controlled studies (RCTs) indexed in FIT‐Nursing Care. We used the platform‐specific filter for “Intervention studies” which displayed 518 studies. We then purposively selected 20 studies which were published on the platform after 2015 as a convenience sample.

#### 2.2.2 Data Comparison

##### 2.2.2.1 Elicit Extractions

We uploaded the studies as PDFs into Elicit and extracted the following variables: study objectives, sample characteristics, study design, participant count, intervention, outcome measured, and intervention effects [5]. Two variables of Elicit were manually adjusted so that the results could be compared with those of FIT‐Nursing Care. The commands behind the columns from Elicit are shown in the Appendix. To examine the demographic characteristics of the participants included in the study, the “sample characteristics” column was derived from the “Population Characteristics” column. We extended the command so that Elicit also extracts, total size of the final sample and a demographic description. Also, to extract the control intervention, we adapted the “Intervention” column so that Elicit also extracts information on the control intervention. We downloaded the data extracted from Elicit in a Comma‐Separated Values (CSV) file and subsequently opened it in an Excel spreadsheet. We conducted the data extraction over a 2‐week period between mid‐August and September 2024.

##### 2.2.2.2 Human Extractions (Reference)

FIT‐Nursing Care follows a defined methodological approach. Two reviewers extract the relevant information for the variables described here and more. The first reviewer fills in all predefined study fields (such as background, study design, etc.) and a second reviewer double‐checks all the information. All reviewers are trained nursing or health researchers [4]. We compared the variables listed in Table 1. The German variables from the RCTs extracted by a human reviewer in the FIT‐Nursing Care platform were manually transferred to the same Excel spreadsheet.

**Table 1:** Variables of Elicit and FIT‐Nursing Care.

Variables in Elicit	Variables in FIT‐Nursing Care
Study objectives	Fragestellung/Zielsetzung
Sample characteristics	Stichprobenbeschreibung
Participant count	Stichprobengrösse
Study design	Design
Interventions	Intervention und Kontrolle
Outcome measured	Primäres Zielkriterium
Intervention effects	Ergebnisse

##### 2.2.2.3 Data Analysis

**Figure 1:**
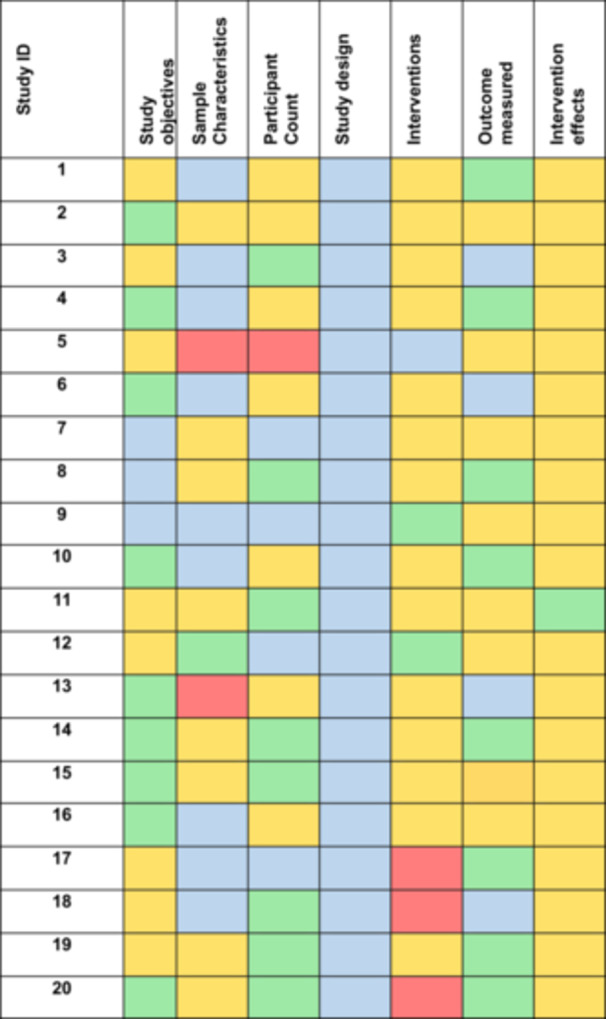
Heatmap of the 7 variables across 20 RCTs compared with the data extraction of Elicit versus human reviewer. Blue = “more,” green = “equal to,” orange = “partially equal” with less information, and red = “deviating.”

We analyzed the combined data set from data extractions using Elicit and FIT‐Nursing Care. One person (J. B.) assessed data completeness and accuracy and classified to: “more,” “equal to,” “partially equal,” and “deviating.” The category “more” meant that Elicit extracted more information than a human reviewer which the human had omitted but was correct. The second category “equal to” meant that Elicit extracted equal information to the human reviewer. “Partially equal” meant that Elicit extracted some equal information, but some important data is missing. The fourth category “deviating” meant that Elicit extracted different/wrong information than a human reviewer. The categories were defined within the study team in a preliminary assessment of five trials which were included in the final sample. The classification was verified by a second person (J. V.). Finally, J. B. and J. V. discussed the “deviating” and “partially equal” categories to assess the extent of the deviation. During the peer review of our article, the fourth category, described as “more,” was established. One author (J. B.) adapted the data extraction table and the results tables. These adjustments were then reviewed by another reviewer (J. V.). We narratively and visually summarized the result (Figure 1). To enhance the transparency of our results, we created a table (see Supporting Information S2: Appendix 2: Table 2 [6, 7, 8, 9, 10, 11, 12, 13, 14, 15, 16, 17, 18]), which presents the categories with one to two examples of each.

## 3 Results

We compared data extractions from *N* = 20 RCTs from 14 different countries (Germany, Argentina, Brazil, South Africa, Austria, Australia, United States of America, England, Norway, Turkey, China, Switzerland, New Zealand, and Italy), published between 2016 and 2021. The topics of the included studies were diverse: maternal, infant and child health, vaccination and infection prevention, mental health and psychoeducation, nurse‐led interventions and self‐management, cancer care and survivorship, therapy and rehabilitation in older adults, symptom management and patient comfort, and health decision‐making and screening. The details of the individual studies are shown in Supporting Information S1: Appendix 1.

Data extractions were “more” by Elicit compared to human extractions in 29.3% of the 7 variables over all 20 studies, “equal to” in 20.7%, “partially equal” in 45.7%, and “deviating” in 4.3% (see Figure 1 and Supporting Information S1: Appendix 1).

For the variable “study objectives,” Elicit extracted “more” compared to human extractions in 15% (3/20 studies), “equal to” in 45% (9/20 studies), “partially equal” with less information being provided in 40% (8/20 studies).

Regarding the variable “sample characteristics,” Elicit was found to have extracted “more” than a human reviewer in 45% (9/20 studies), “equal to” in 5% (1/20 studies), “partially equal” in 40% (8/20 studies), and in 10% of studies (2/20 studies) the data were “deviating” from that extracted by human reviewer.

For the variable “participant count,” Elicit extracted “more” than a human reviewer in 20% of studies (4/20 studies). In 40% (8/20), “equal to” information was provided by Elicit. In 35% of studies, the data extracted by Elicit was “partially equal” with less information and 5% (1/20 studies) was “deviating” from the data extracted by the human reviewer.

For the “study design” variable, Elicit extracted “more” in all studies (100%) than a human reviewer.

For the variable “Interventions,” Elicit's data extraction was in 5% (1/20 studies) “more” than by the human reviewer, “equal to” in 10% (2/20 studies), “partially equal” with less information in 70% (14/20 studies), and in 15% (3/20 studies) “deviating.”

For “outcome measured,” the results demonstrated that in 20% of studies, Elicit extracted “more” data than that by the human reviewer. In 40%, the data were found to be “equal to” and in 40%, the data were found to be “partially equal” with less information available.

For the variable “intervention effects,” 5% of the studies (1/20 studies) “more” data were extracted by Elicit than by human reviewer; the remaining 95% were “partially equal” with less information.

## 4 Discussion

Our aim was to compare data extraction from Elicit versus a human reviewer from FIT‐Nursing Care. Overall, in just below half of the variables which were compared Elicit extracted data that was “partially equal.” In one‐third of the data extraction, Elicit extracted “more” than the human reviewer. Only 4.3% of the extracted data from Elicit “deviated” from the human reviewer.

The process of extracting data from full texts is inherently labor‐intensive. Furthermore, the inconsistent application of extraction criteria across studies and human reviewers represents another source of variability. Additionally, the process of information retrieval is subject to variability in interpretation, which may also impact the accuracy of reported effects. As with any process involving human input, there is always the potential for human error to negatively impact the results [3]. If an AI tool could efficiently, accurately, and completely extract the data required it could facilitate the review process. Several scoping reviews have highlighted the potential of generative AI to assist in the data extraction process [19, 20, 21, 22]. However, Elicit has not been independently evaluated before. Elicit has demonstrated that for certain variables, the information can be extracted accurately, for example, for the study design of RCTs. Elicit demonstrated proficiency, accurately extracting data and providing sufficient information in 100% of the studies. However, this needs to be tested for other study designs in further research. Overall, Elicit extracted 29.3% more than humans across all 7 variables and 20 trials. This may indicate a higher sensitivity and possibly consistency of the system in identifying information. However, this result should be interpreted with caution, as extracting “more” does not necessarily equate to extracting better or more accurate information. It is questionable if the additional information was always needed or indeed relevant. Therefore, further studies should focus on the qualitative evaluation of the extracted content and assess the precision and relevance of the additional data provided by Elicit.

For the variables sample characteristics, interventions, intervention effect, Elicit extracted “partially equal” with less information than human reviewer. However, it is questionable whether a less detailed extraction, as observed for the “intervention effect” variable with only 5% complete agreement, should be rated as “poor.” It is important to note that discrepancies in the extraction process do not necessarily indicate inaccuracy in the results. Rather, they suggest that Elicit has likely prioritized different aspects than the human reviewer. It is essential to consider the context and the specific objective of Elicit's intended use to ascertain its suitability. It is also conceivable that the reviewer responsible for data extraction on FIT‐Nursing Care may identify information of greater relevance than that extracted by Elicit. It is also the case that, even with systematic reviews, different authors extract the data differently, and the level of detail must therefore be determined beforehand.

It is important to note the possibility that the results could be improved by adjusting the command or configuration of Elicit. This is exemplified by the variable “Outcome measured,” where in 20% “more” data were extracted and in 40% “equal to” and in 40% “partially equal” with less information. It is possible that targeted adjustments to the command underlying the variable “outcome measured” in Elicit could increase precision, thereby significantly enhancing the utility of the tool. However, this was not tested as part of our project. In their mapping review, Cierco Jimenez et al. [22] describe various AI‐supported tools that can be used during the SR process. However, the evaluation of the accuracy and precision of these tools is missing. Our findings are similar to previous studies analyzing the performance of AI‐supported tools. Lieberum et al. [19] show in their scoping review several tools, such as Generative Pretrained Transformer (GPT) and Claude, to support the data extraction process. Blaizot et al. [21] also demonstrated in their SR, several AI‐supported tools that could facilitate the creation of an SR. For instance, the software SWIFT‐Review is used for data extraction, but the software was not able to automate all aspects of data extraction. Consequently, individual variables, such as the sample size, had to be entered manually. A manual review of the automated processes was also necessary to identify any missing information in the data extraction from the software [21]. The results in the SR from Marshall and Wallace [20] suggest that AI‐supported data extraction tools for systematic review—such as ExaCT and RobotReviewer—have made notable progress, but are still at an early stage of development. Although these tools have demonstrated encouraging accuracy rates, they are not yet sufficiently accurate to fully replace manual data extraction. The performance of these tools is constrained by limited and often imperfect training data, which can reduce accuracy and reliability [20].

Further, it is important to note that Elicit is currently unable to extract information from figures, such as flowcharts, which represents a significant limitation. Five of the involved studies used visual data in the form of charts, graphs, or tables [23, 24, 25, 26, 27]. Consequently, data extraction must be conducted manually if information from tables and figures needs to be extracted. It is also important to note that Elicit is subject to ongoing development, with new features being added frequently. Since conducting our study, Elicit has added the following features at the start of 2025: for example, extract data from tables in papers with high accuracy columns and is likely to amend their product continuously.

### 4.1 Limitations

First, the studies from FIT‐Nursing Care were used as a reference, having been extracted by different human reviewers. Even though these humans were educated, and data were double‐checked, we cannot exclude that different reviewers extract data in different levels of detail. It must be acknowledged that human data extraction is not always flawless and needs to be performed independently in a rigorous approach. Second, the data from FIT‐Nursing Care were in German and the data from Elicit were in English. So, there was a comparison between German and English data extraction which could have led to translation errors which, however, is unlikely as the native language of the authors is German and all are proficient in English. Furthermore, we assessed a convenience sample of 20 RCTs and further studies need to confirm our results within a larger sample size.

## 5 Conclusion

Elicit was capable of extracting data (partly) correct for our predefined variables, particularly for variables such as the study design. However, it is important to consider the limitations of the tool, particularly in terms of its ability to process detailed data and visual information. In the extraction of more complex data, such as the variables “intervention effects” or “intervention,” Elicit demonstrated to have certain deficits. Thus, human verification is necessary to ensure that all relevant information is captured completely and correctly by Elicit but future studies are needed to confirm our results. The combination of automated data extraction and human control might enhance efficiency while maintaining scientific accuracy.

## 6 Implications

Systematic reviews are labor‐intensive. The data extraction process may be facilitated by AI tools. In 29.3%, Elicit extracted “more” data, and in 20.7%, “equal to” information compared to a human reviewer. However also half of the data showed “partially equal” data or “deviating” data. Use of Elicit may require a human reviewer to double‐check the extracted data. AI tools may facilitate the data extraction process during synthesis. Elicit may be an important tool for other research fields than health.

## Author Contributions

**Joleen Bianchi:** conceptualization, writing – original draft, writing – review and editing. **Julian Hirt:** conceptualization, writing – review and editing, writing – original draft, supervision. **Magdalena Vogt:** conceptualization, writing – original draft, writing – review and editing. **Janine Vetsch:** conceptualization, writing – review and editing, writing – original draft, supervision.

## Ethics Statement

The authors have nothing to report.

## Conflicts of Interest

The authors declare no conflicts of interest.

## Clinical Resources

- Elicit: The AI Research Assistant, https://elicit.com/ [5].
- FIT Nursing Care, Knowledge Plattform for Nurses in the German‐speaking area, https://www.fit-care.ch.

## Supporting information

Appendix 1: Raw Data Extraction.

Appendix 2: Table with examples from the analysis.

Appendix 3 Description of the commands by Elicit.

Strobe Checklist.

## Data Availability

All data supporting the results of this study are available in the article or in the Appendix.
