# Supplementary material for: Data Extractions Using a Large Language Model (Elicit) and Human Reviewers in Randomized Controlled Trials: A Systematic Comparison
Source: Cochrane Evid Synth Methods. 2025 Jun 8;3(4):e70033. doi: 10.1002/cesm.70033 (PMC12462964; doi:10.1002/cesm.70033)
Supplement: Supplementary file 2 — Appendix 2: Table with examples from the analysis. [file CESM-3-e70033-s002.docx]

**Appendix 2:** Table 2 Results of the comparative analysis of Elicit vs. FIT-Nursing Care

| **Variables** | **Category** | **% (number/n)** | **References** | **Examples Elicit** | **Examples Human (FIT)** |
| --- | --- | --- | --- | --- | --- |
| **Study objectives** | more | 15 (3/20) | Gerçeker et al. (2018) | The main objectives of the study were to compare the impact of standardized flushing methods using ANTT with single-use pre-filled flush syringes versus manually prepared syringes on CVC occlusion rates and CLABSI rates in PHO patients. | Das Ziel war der Vergleich von zwei standardisierten Spülmethoden mit der aseptischen non-Touch-Technik zum Spülen eines Zentralen Venenkatheter; die manuell vorbereiteten Spritzen im Vergleich mit einer vorgefüllte Spülspritzen zum Einmalgebrauch. |
|  |  |  | Garnæs et al. (2016) | - Primary objective: Assess whether regular supervised exercise training in pregnancy could reduce gestational weight gain (GWG) in women with prepregnancy overweight/obesity.  - Secondary objectives: Examine the effects of exercise in pregnancy on 30 outcomes including gestational diabetes mellitus (GDM) incidence, blood pressure, blood measurements, skinfold thickness, and body composition | Ziel war zu untersuchen, ob ein regelmässiges und supervisiertes Bewegungstraining bei schwangeren Frauen mit einem BMI von mind. 28kg/m2 im Vergleich zur Standardversorgung übermässige Gewichtszunahmen reduzieren und andere klinische Zielkriterien verbessern kann. |
|  | Equal to | 45 (9/20) | Raphaelis et al. (2020) | - Describe recruitment and characteristics of the target population (Reach)  - Report on overall effectiveness of the ANtiPain intervention (Effectiveness)  - Determine which elements of implementation may play a role on the effectiveness of the ANtiPain intervention (Implementation) | Spezifische Ziele der Studie sind: (1) Beschreibung der Rekrutierung und Merkmale der Zielpopulation (Reichweite); (2) Berichterstattung über die Gesamtwirksamkeit der Intervention (Wirksamkeit) und (3) welche Elemente der Umsetzung eine Rolle für die Wirksamkeit der Intervention spielen können (Umsetzung). |
|  |  |  | Glass et al. (2017) | - Compare safety and mental health outcomes between women using the tailored Internet-based safety decision aid and a control group  - Assess if the intervention reduces decisional conflict, increases safety behaviors, and reduces repeat IPV exposure (primary outcomes)  - Assess if the intervention reduces depression and PTSD symptoms (secondary outcomes) | Ziel war, die Wirksamkeit einer internetbasierten Entscheidungshilfe bzgl. Sicherheit für von Gewalt in der Partnerschaft betroffene Frauen im Vergleich mit einer Informations-Website hinsichtlich Sicherheit und psychologischen Zielkriterien über zwölf Monate zu untersuchen; Hypothese war, dass die Intervention Entscheidungskonflikte, wiederholte Gewalt in der Partnerschaft, Depression und posttraumatische Belastungsstörung reduziert und das Sicherheitsverhalten verbessert. |
|  | Partially equal | 40 (8/20) | Polack et al. (2020) | Assess the safety and reactogenicity of the BNT162b2 vaccine, including local and systemic adverse events and use of antipyretic/pain medication  -Evaluate the efficacy of the BNT162b2 vaccine against confirmed COVID-19 cases, both in participants with and without prior SARS-CoV-2 infection  -Evaluate the efficacy of the BNT162b2 vaccine against severe COVID-19 cases | Untersucht wurde die Sicherheit und Wirksamkeit von zwei 30-μg-Dosen von BNT162b2, die im Abstand von 21 Tagen intramuskulär verabreicht wurden, im Vergleich zu Placebo. |
|  |  |  | Rock et al. (2015) | Primary objective: Measure weight loss at 1 and 2 years  - Secondary objective: Explore factors that may modify weight loss at 24 months (e.g. time since diagnosis, tumor type, therapy)  -Hypothesis: The behavioral weight loss intervention would lead to greater weight loss compared to the control group | Ziel war, die Wirksamkeit einer Intervention zur nachhaltigen Gewichtsabnahme und Verhaltensänderung bei übergewichtigen Frauen nach einer Brustkrebsdiagnose mit gesteigerter körperlicher Aktivität und reduzierter Energieaufnahme zu untersuchen. Hypothese war, dass eine verhaltensbezogene, individuell zugeschnittene Intervention mit Schwerpunkt auf gesteigerter körperlicher Aktivität, der Standardbetreuung überlegen ist hinsichtlich der Gewichtsabnahme |
| **Sample characteristics** | more | 45 (9/20) | Della Forster et al. (2017) | '- A total of 635 women were recruited and randomly assigned, with 319 in the antenatal expressing group and 316 in the standard care group.  - After exclusions, 632 infants were available for the primary analysis, with 317 in the antenatal expressing group and 315 in the standard care group.  - Most women in both groups had gestational diabetes, more than half were expecting their first baby, and half were either overweight or obese | 635 Frauen (319 in der Interventions-, 316 in der Kontrollgruppe), die meisten davon waren verheiratet oder lebten in einer Partnerschaft, die meisten hatten einen Gestationsdiabetes, mehr als die Hälfte der Frauen war mit dem ersten Kind. |
|  |  |  | Volk et al. (2020) | A total of 516 participants were recruited for the study  - 370 (71.7%) were younger than 65 years  - 320 (62.0%) were female  - 138 (26.7%) identified as Black  - 47 (9.1%) did not have health insurance  - 226 (43.8%) had a high school or lower educational level  - 227 participants were randomized to the PDA group and 224 to the EDU group | Es wurden 516 Teilnehmende in die Analyse eingeschlossen (Interventionsgruppe: n=259, Kontrollgruppe: n=257). Die Teilnehmenden waren vorwiegend unter 65-jährig (71.7 %) und weiblich (62.0 %). 43.8 % hatten einen High School Abschluss oder einen tieferen Bildungsabschluss. Die Charakteristika der Teilnehmenden waren zwischen den Gruppen vergleichbar |
|  | Equal to | 5 (1/20) | Polack et al. (2020) | A total of 43,548 participants were recruited, with 43,448 receiving either the BNT162b2 vaccine (21,720 participants) or placebo (21,728 participants).  - 49% of the 37,706 participants with at least 2 months of safety data were female.  - 83% were White, 9% were Black or African American, and 28% were Hispanic or Latinx.  - 35% were obese (BMI ≥ 30) and 21% had at least one coexisting condition.  - The median age was 52 years, and 42% were older than 55 years of age. | Stichprobenbeschreibung:  Zwischen dem 27. Juli 2020 und dem 14. November 2020 wurden insgesamt 44 820 Personen gescreent, und 43 548 Personen im Alter von 16 Jahren oder älter wurden an 152 Standorten weltweit (USA, 130 Standorte; Argentinien, 1; Brasilien, 2; Südafrika, 4; Deutschland, 6; und Türkei, 9) im Phase-2/3-Teil der Studie randomisiert.  Insgesamt erhielten 43 448 Personen entweder den Impfstoff (21 720) oder Placebo (21 728). Zum Stichtag am 9. Oktober hatten insgesamt 37 706 Personen im Median von mindestens zwei Monaten Daten zur Sicherheit des Impfstoffes nach der zweiten Dosis zur Verfügung. Von diesen 37 706 Personen waren 49% weiblich, 83% waren weiss, 9% waren schwarz oder afroamerikanisch, 28% waren hispanisch oder lateinamerikanisch, 35% waren übergewichtig (Body-Mass-Index von mindestens 30.0) und 21% hatten mindestens eine Begleiterkrankung. Das Durchschnittsalter betrug 52 Jahre und 42% der Personen waren älter als 55 Jahre. |
|  | Partially equal | 40 (8/20) | Glass et al. (2017) | '- A total of 1,072 participants were screened, with 865 (80.7%) being eligible  - 841 (97.2%) of the eligible participants consented and were randomized, with 418 in the intervention group and 423 in the control group  - 725 participants completed baseline measures, with 365 in the intervention group and 360 in the control group  - The sample was diverse in age, with many participants living with their abuser and having children at home  - The majority of abusers were male and current partners/spouses | Es wurden 841 durchschnittlich 33-jährige Teilnehmende mit eher niedrigem Bildungsstand und mit einem hohen Risiko für häusliche Gewalt eingeschlossen (IG: n=418; KG: n=423), von denen mehr als die Hälfte mit ihren überwiegend männlichen Partnern und ca. jede vierte Teilnehmerin mit unter 18-jährigen Kindern zu Hause zusammenlebten.  Die Teilnehmenden besuchten die Website am häufigsten von zu Hause aus; eher selten bei der Arbeit, bei Bekannten oder in öffentlichen Orten. |
|  |  |  | Yıldırım et al. (2019) | - A total of 204 patients were recruited  - 102 patients were in the experimental group and 102 were in the control group  - The majority were male  - The average age was 61 ± 14 years  - Most were married and had a primary school education level  - Most had inadequate income levels and were unemployed | Es wurden 204 Teilnehmende in die Analyse eingeschlossen, welche gleichermassen auf die Interventions- und Kontrollgruppe verteilt waren. Die Mehrheit der Teilnehmenden war männlich, das Durchschnittsalter lag bei 61 Jahren (Standardabweichung: ± 14). Die soziodemografischen Merkmale sowie die Opioiddosen der Teilnehmenden unterschieden sich nicht statistisch signifikant voneinander. In der Interventionsgruppe war die Anzahl derer, die nicht täglich Stuhlgang verrichteten, statistisch signifikant niedriger als in der Kontrollgruppe, während die Anzahl derer, die einmal oder mehrmals täglich Stuhlgang hatten, in der Interventionsgruppe höher war (p < 0.05). |
|  | deviated | 10 (2/20) | Taylor et al. (2017) | - A total of 802 pregnant women were recruited, with 58.1% of those eligible participating.  - The participants were mostly European (85%), with 48% having their first child and 41% being overweight or obese before pregnancy.  - Participants were older, had less household deprivation, and were more likely to identify as European compared to those who did not consent to participate.  - Retention was high at 2 years, with those remaining being older, less likely to be Māori or Pacific, more highly educated, and from less deprived households. | Es wurden 847 Teilnehmende eingeschlossen (Gruppe FAB: n=214, Gruppe Kombination: n=210, Gruppe Schlaf: n=209; Kontrollgruppe: n=214). Für 48 % war es das erste Kind und 41 % der Schwangeren waren bereits vor der Schwangerschaft übergewichtig. |
|  |  |  | Rickard et al. (2021) | A total of 2944 adult and pediatric patients were recruited, with 2941 included in the mITT analysis  - 1463 patients were assigned to the 7-day infusion set replacement group  - 1481 patients were assigned to the 4-day infusion set replacement group (control)  - Patients had various types of central venous access devices or peripheral arterial catheters, and were from medical, surgical, cancer, and intensive care units (except neonatal ICUs) | Von 6007 Patientinnen und Patienten wurden 2941 in die mITT Analyse aufgenommen (Interventionsgruppe: n= 1124 zentralvenöse Katheter, n= 357 periphere arterielle Katheter; Kontrollgruppe; n= 1097 zentralvenöse Katheter, n= 363 periphere arterielle Katheter  Bei der Interventionsgruppe waren es 1293 Erwachsene (87.3%) mit einem Durchschnittsalter von 59 Jahren und 188 Kinder (12.7%) mit einem Durchschnittsalter von 2.3 Jahren, wobei die Mehrheit (63.1%) männlich war.  Bei der Kontrollgruppe waren es 1259 Erwachsene (86.2%) mit einem Durchschnittsalter von 57 Jahren und 201 Kinder (12.7%) mit einem Durchschnittsalter von 3.2 Jahren, wobei die Mehrheit (62.7%) männlich war.  Beschreibung der Baseline Charakteristik anhand des Alters, verbrachte Hospitalisationstage vor Kathetereinlage, Geschlecht, Diagnose, Stationszugehörigkeit, Kathetertyp, Zugangsstelle, Lumen, Fachperson bei Einlage und Anzahl der Versuche, Einlageort, erhaltene Antibiose und Heparininfusionen. |
| **Participant count** | more | 20 (4/20) | Baden et al. (2021) | The total participant count in this study is 30,420, with 15,210 participants randomized to each group (placebo and mRNA-1273 vaccine). | Stichprobengrösse:  Angaben zur Stichprobenberechnung im Protokoll ersichtlich |
|  |  |  | Gerçeker et al. (2018) | The total number of participants in the study was 48, with 23 in the intervention group and 25 in the control group. | Es wurden 48 Personen eingeschlossen |
|  | Equal to | 40 (8/20) | Lay et al. (2018) | The total number of participants randomized at baseline was 238, with 119 in the intervention group and 119 in the TAU group. | Stichprobengrösse: n=238 (I: 119, K:119); Studienpartizipation, Ausfallrate wurde in Flussdiagramm dargestellt |
|  |  |  | Gerçeker et al. (2018) | The total number of participants in the study was 48, with 25 in the intervention group and 23 in the control group. | Es wurden insgesamt 48 Kinder beider Geschlechter mit ihren Müttern in die IG (n=25) und KG (n=23) eingeschlossen |
|  | Partially equal | 35 (7/20) | Volk et al. (2020) | The total number of participants enrolled in the study was 516. | Es wurden 516 Teilnehmende in die Analyse eingeschlossen (Interventionsgruppe: n=259, Kontrollgruppe: n=257). |
|  |  |  | Leininger et al. (2016) | The total number of participants in the study was 241. | Es wurden insgesamt 241 (SA I: n=79; SA II: n=80; SA III: n=82) rekrutiert |
|  | deviated | 5 (1/20) | Rickard et al. (2021) | The total number of participants randomized at baseline was 2944, with 1463 in the 7-day group and 1481 in the 4-day group. | 1. Von 6007 Patientinnen und Patienten wurden 2941 in die mITT Analyse aufgenommen (Interventionsgruppe: n= 1124 zentralvenöse Katheter, n= 357 periphere arterielle Katheter; Kontrollgruppe; n= 1097 zentralvenöse Katheter, n= 363 periphere arterielle Katheter |
| **Study design** | more | 100 (20/20) | Leininger et al. (2016) | The study design was a randomized controlled trial (RCT) conducted at a single site. It was not double-blind, as the participants and providers were aware of the treatment assignments. The study used both a societal and healthcare perspective in the cost-effectiveness analysis. | Randomisiert kontrollierte Studie (RCT) |
|  |  |  | Rickard et al. (2021) | The study design was a randomized, controlled, equivalence and non-inferiority trial with assessor-masked primary and secondary outcomes. It was conducted at 10 hospitals in Australia and used stratified randomization to allocate patients to either 7-day or 4-day infusion set replacement. | Interventionsstudie (kontrolliert, randomisiert)  Definition des zeitlichen Ablaufs: prospektiv |
| **Interventions** | more | 5 (1/20) | Rickard et al. (2021) | 1. Infusion set replacement every 7 days (intervention group)  2. Infusion set replacement every 4 days (control group)  The infusion sets included various fluids and medications, but excluded lipids, inotropes, chemotherapy, cyclosporin, and blood products. | Intervention: Wechselintervall der Infusionssets alle 7 Tage  Kontrolle: Wechselintervall der Infusionssets alle 4 Tage |
|  | Equal to | 10 (2/20) | Gerçeker et al. (2018) | 1. Standardized flushing method with ANTT using single-use pre-filled flush syringes (intervention group)  2. Standardized flushing method with ANTT using manually prepared syringes (control group)  The flushing procedures for both groups included the pulsatile technique, use of 10-mL syringe size with 0.9% NaCl, flushing once a day, and flushing training for the nurses.  Additionally, a care bundle for preventing catheter infection was applied in the unit, which included measures related to catheter entries, site care, dressing/site assessment, and catheter cap/hub/tubing care. | Intervention: Fertigspritzen  Für die Katheterspülung wurden eine standardisierte Spülmethode mit non-Touch-Technik unter Verwendung von Fertigspritzen zum Einmalgebrauch verwendet.  Die in der Interventions- und Kontrollgruppe verwendeten Schritte der Katheterspülung werden in dieser Studie beschrieben.  Die standardisierte Spülmethode umfasste die pulsatile Technik (Push-Pausen-Technik), die Verwendung einer 10-ml-Spritze (Goldstandard beim Spülen von ZVK) mit 0.9 % NaCl zum Spülen, einmal täglich durchspülen und das Training der Pflegepersonen zum Thema spülen.  Kontrolle: manuell hergestellte Spritzen  Für die Katheterspülung wurden eine standardisierte Spülmethode mit non-Touch-Technik unter Verwendung von manuell hergestellten Spritzen verwendet. |
|  |  |  | Polack et al. (2020) | 1. Participants were randomly assigned in a 1:1 ratio to receive either the BNT162b2 vaccine or a placebo.  2. Both groups received two doses of their assigned intervention, 21 days apart.  3. The dose of the BNT162b2 vaccine was 30 μg.  4. The BNT162b2 vaccine is a lipid nanoparticle-formulated, nucleoside-modified RNA vaccine that encodes the SARS-CoV-2 spike protein. | Interventionsgruppe: Intramuskuläre Verabreichung von zwei 30-μg-Dosen BNT162b2 im Abstand von 21 Tagen.  BNT162b2 enthält Lipid-Nanopartikel mit modifizierter mRNA, welche für das Spike-Protein des Virus kodiert.  Kontrollgruppe: Intramuskuläre Verabreichung von zwei Dosen Placebo im Abstand von 21 Tagen. |
|  | Partially equal | 70 (14/20) | Baden et al. (2021) | 1. mRNA-1273 vaccine: Two intramuscular injections of 100 μg, administered 28 days apart  2. Placebo: Saline injections administered in the same way as the vaccine | Interventionsgruppe:  Injektion des mRNA-1273-Impfstoff in den Deltamuskel. Die Injektionen erfolgten im Abstand von 28 Tagen in denselben Arm (100 μg mRNA-1273).  Der mRNA-1273-Impfstoff ist ein mit Lipid-Nanopartikeln verkapselter mRNA-basierter Impfstoff.  Kontrollgruppe:  Gleiches Vorgehen wie bei der Interventionsgruppe, jedoch mit Kochsalzlösung als Placebo. |
|  |  |  | Moadad et al. (2016) | The main intervention in this study was the use of the "BUZZY" device, which combines cold and vibration stimulation. The "BUZZY" was applied 5-10 cm proximal to the IV insertion site and remained in place and vibrating throughout the procedure. Participants were randomly assigned to either the "BUZZY" intervention group or the control group that did not receive the "BUZZY". | Interventionsgruppe (IG): PVVK-Einlage unter Einsatz des Buzzys®  Der batteriebetriebene Buzzy® in Form einer Plastik-Biene im Umfang von 8 x 5 x 2.5 cm kombiniert Kälte und Vibration und wird proximal der Punktionsstelle fixiert um die Schmerzwahrnehmung zu reduzieren.  Die Kinder und Eltern wurde die Möglichkeit gegeben während der PVVK-Einlage (24G) den Buzzy® zu halten und die Vibration einzuschalten; kurz vor dem Punktionsversuch legte die Pflegende ein Ice-Pack unter das Gerät und wendete dies 5–10 cm proximal des Handrückens an. Die Kinder wurden aufgefordert sich auf die Wahrnehmung des Buzzys® zu konzentrieren und nicht der Punktion zu folgen.  Kontrollgruppe (KG): Standardversorgung  PVVK-Einlage ohne Einsatz des Buzzys® |
|  | deviated | 15 (3/20) | Sorrentino et al. (2017) | The interventions in this study were the use of 6 different automated devices for capillary blood collection in newborns: Ames Minilet™ Lancet, Cardinal Health Gentleheel ®, Natus Medical NeatNick™, BD Quikheel™ Lancet, Vitrex Steriheel ® Baby Lancet, and Accriva Diagnostics Tenderfoot ®. All devices used a trigger and spring loaded mechanism to perform an automatic heel stick. | Intervention:  Alle Geräte lösten automatisch einen Fersenstich aus, der zuerst einen Hautschnitt bewirkte und sich anschliessend von selbst in sein Gehäuse zurückzog, um die Gefahr einer Nadelstichverletzung zu minimieren.  Alle Neugeborenen erhielten unter Berücksichtigung des institutionellen Protokolls zur Schmerzkontrolle oral einen Milliliter 10 %-ige Glukose zwei Minuten vor der Blutabnahme zur nicht pharmakologischen Schmerzprävention.  Es wurden folgende 5 Geräte mit dem Kontrollapparat verglichen:  Cardinal Health Gentleheel ®: Bogenförmiger Hautschnitt, einseitig abgeschrägte Klinge  Natus Medical NeatNick™: Bogenförmiger Hautschnitt, einseitig abgeschrägte Klinge,  BD Quikheel™ Lancet: Im 90° Grad Winkel nach unten gerichteter Druck, einseitig abgeschrägte Klinge  Vitrex Steriheel ® Baby Lancet: Bogenförmiger Hautschnitt, einseitig abgeschrägte Klinge  Accriva Diagnostics Tenderfoot ®: Bogenförmiger Hautschnitt, doppelt abgeschrägte und geschliffene Klinge  Kontrolle:  Ames Minilet™ Lancet: Im 90° Winkel nach unten gerichteter Druck |
|  |  |  | Glass et al. (2017) | 1. A priority-setting activity where participants made pairwise comparisons of factors related to IPV safety decisions and received feedback on their priorities.  2. Completion of the Danger Assessment (DA) or DA-Revised (DA-R) to assess their risk level, with visual feedback and messages about their danger level.  3. Tailored safety action plans with recommended strategies based on their input and local resources.  The control group received the same emergency safety plans as the intervention group, but did not receive the priority-setting activity, DA/DA-R feedback, or tailored safety action plans. | Intervention: Internetbasierte Entscheidungshilfe bzgl. Sicherheit  Die Online-Aktivitäten konnten zu einem frei wählbaren und sicheren Zeitpunkt erfolgen; wurden die Aktivitäten nicht abgeschlossen, wurden die Teilnehmenden telefonisch und/oder per E-Mail daran erinnert.  Die Online-Aktivitäten beinhalteten Fragen zur Priorisierung von Entscheidungstreibern hinsichtlich ihres Sicherheitsverhaltens mit Feedback dazu sowie ein Assessment zu Risikofaktoren für schwere Gewalt (Danger Assessment bzw. DA-R); Entscheidungstreiber sind z. B. verfügbare Ressourcen, Gefühle für die Partnerin bzw. den Partner und Sicherheitsbedenken.  Zudem waren Notfall-Sicherheitspläne und Hinweise auf andere Unterstützungsangebote Teil der Intervention; diese beinhalteten konkrete Aktionspläne und massgeschneiderte Empfehlungen.  Basierend auf den beschriebenen Interventionsbestandteilen entwickelten die Teilnehmenden einen individuellen Sicherheitsplan, den sie jederzeit online oder ausgedruckt zur Verfügung hatten.  Kontrolle: Informations-Website  Die Informations-Website hat die gleichen Notfall-Sicherheitspläne wie die Intervention zum Inhalt |
| **Outcome measured** | more | 20 (4/20) | Taylor et al. (2017) | 1. BMI at 24 months  2. BMI-for-age z score at 24 months  3. Prevalence of obesity at 24 months | Primäre Zielkriterien:  BMI und Gewicht des Kleinkindes nach Vollendung des 2. Lebensjahr |
|  |  |  | Rock et al. (2015) | The primary outcome measured in this study was weight loss, measured as a percentage of initial weight at 12 and 24 months. | Primäre Zielkriterien:  Gewicht (kg)  Gewichtsveränderung (%) |
|  | Equal to | 40 (8/20) | Glass et al. (2017) | 1. Decisional conflict, as measured by the Decision Conflict Scale  2. Safety behaviors, as measured by the number of safety behaviors used and how helpful they were  3. Repeat IPV exposure, as measured by the Severity of Violence Against Women Scale | Primäre Zielkriterien:  Entscheidungskonflikte: Decision Conflict Scale  Sicherheitsverhalten: Adaptierte Liste mit 35 Items zum Sicherheitsverhalten  Gewalt-Exposition: Severity of Violence Against Women Scale und Women's Experience of Battering Scale |
|  |  |  | Sorrentino et al. (2017) | 1. Number of punctures performed to collect blood  2. NIPS (Neonatal Infant Pain Scale) score  3. The need to squeeze the heel (defined as pressure on the heel to increase blood flow) | Primäre Zielkriterien:  Anzahl der Punktionen für die Blutabnahme  Prozeduraler Schmerz während der Blutabnahme: Neonatal Infant Pain Scale (NIPS)  Notwendiger Druck auf die Ferse, um die Durchblutung zu erhöhen |
|  | Partially equal | 40 (8/20) | Yıldırım et al. (2019) | 1) Constipation severity, feeling of incomplete bowel emptying, straining severity, anal pain severity, and bloating  2) Stool consistency  3) Number of defecations  4) Quality of life | Zielkriterien und Messinstrumente: Baseline-Daten wurden zur Diagnose, zu den Medikamenten, Laxantien, zur Flüssigkeitsaufnahme, zur körperlichen Betätigung, zum Opioidkonsum, zur wöchentlichen Ernährung, zur Bewegung/zum Aktivitätsniveau, zur Menge der aufgenommenen Ballaststoffe (Gramm pro Tag) und zu den Eigenschaften des Stuhlgangs erhoben. Die weitere Datenerhebung erfolgte ab der zweiten Woche nach Beginn der Opioidbehandlung und während der folgenden fünf Wochen bei jedem Stuhlgang:  Eigenschaften des Stuhls und Symptome von Obstipation: Defäkationstagebuch  Stuhlform: Bristol stool scale  Intensität von Obstipationssymptomen: Visual analogue scale (VAS)  Auswirkungen der Obstipation auf die Lebensqualität: Patient assessment of constipation of quality of life scale (PACQLQ) |
|  |  |  | Lay et al. (2018) | The primary outcome measured in this study is the number of compulsory readmissions to psychiatric inpatient care over the 24-month period. | Primäre Zielkriterien: Re-Hospitalisierungsrate (freiwillig und zwangsweise) sowie Dauer der Klinikaufenthalte zwischen Klinikaustritt und Zeitpunkt der letzten Datenerhebung (t2) |
| **Intervention effects** | Equal to | 5 (1/20) | Lay et al. (2018) | - Proportion of participants with compulsory readmission: 28% in intervention group vs. 43% in TAU group  - Number of compulsory readmissions per patient: 0.6 in intervention group vs. 1.0 in TAU group  - Length of compulsory hospital episodes: 15 days in intervention group vs. 31 days in TAU group  - Relative risk of compulsory readmission: 0.6 (95% CI 0.3-0.9) for intervention group vs. TAU group  All of these differences were statistically significant, indicating the intervention program was effective in reducing compulsory psychiatric readmissions compared to standard care. | Primäre Zielkriterien:  Anzahl psychiatrische Re-Hospitalisationen, Anzahl Hospitalisationstage.  75 Probanden der Interventions- und 93 Probanden der Kontrollgruppe vollendeten das 24-Monate-Programm.  Die Rate wiederholter Zwangseinweisungen der Interventionsgruppe (28%) war bei Studienabschluss signifikant tiefer als jene der Kontrollgruppe (43%) (p = 0.038). Die Aufenthaltsdauer bei Zwangseinweisungen war bei der Interventionsgruppe signifikant kürzer (15 Tage) als bei der Kontrollgruppe (31 Tage) (p = 0.047). Probanden der Interventionsgruppe hatten ein signifikant tieferes Risiko für eine erneute Zwangseinweisung als die Kontrollgruppe (RR = 0.55, p = 0.027). Die Anzahl Zwangseinweisungen in der Vergangenheit wurde in dieser Analyse als grösster Risikofaktor identifiziert (RR = 2.83, p < 0.0001). |
|  | Partially equal | 95 (19/20) | Toots et al. (2016) | No significant between-group differences in ADL independence at 4 or 7 months  - Significant between-group improvement in balance favoring the exercise group at 4 months  - Positive effects of the exercise program on ADL independence and balance in participants with non-Alzheimer's dementia, but not in those with Alzheimer's disease | ATL-Abhängigkeit:  FIM: Sowohl nach vier als auch nach sieben Monaten kein statistisch signifikanter Gruppenunterschied (MD: 1.34, 95 %-KI: -1.56 bis 4.25, p=0.36 bzw. MD: 0.78, 95 %-KI: -2.21 bis 3.77, p=0.61)  Barthel-Index: Sowohl nach vier als auch nach sieben Monaten kein statistisch signifikanter Gruppenunterschied (MD: 0.60, 95 %-KI: -0.24 bis 1.44, p=0.16 bzw. MD: 0.57, 95 %-KI: -0.30 bis 1.43, p=0.20)  Balance: Nur nach vier, aber nicht mehr nach sieben Monaten statistisch signifikanter Vorteil durch die Intervention (4.20, 95 %-KI: 1.79 bis 6.61, p<0.001 bzw. MD: -0.02, 95 %-KI: -2.53 bis 2.49, p=0.98)  Weitere Ergebnisse:  Interaktionseffekte: Bei Teilnehmenden mit Demenz nicht vom Alzheimer-Typ positive Zwischen-Gruppen-Effekte hinsichtlich FIM nach sieben Monaten, BI nach vier Monaten und BBS nach vier und sieben Monaten. Nach sieben Monaten bei Menschen mit Demenz vom Alzheimer-Typ negative Auswirkungen hinsichtlich FIM und BBS.  Subgruppenanalyse: Hinweis auf höhere Wirksamkeit bei besserer Kognition (nur bzgl. BBS, nicht bzgl. FIM und BI)  Unerwünschte Ereignisse: Insgesamt nur kleine und vorübergehende unerwünschte Ereignisse. Bei einem Todesfall kann ein indirekter Zusammenhang zur Intervention nicht mit vollständiger Sicherheit ausgeschlossen werden. |
|  |  |  | Glass et al. (2017) | - Significantly greater reduction in decisional conflict immediately after using the decision aid  - Significantly greater increase in percentage of safety behaviors found helpful over 12 months  - Significantly higher percentage of participants who had ended their abusive relationship by 12 months | Primäre Zielkriterien:  Entscheidungskonflikte: Statistisch signifikanter Vorteil durch die Intervention (Beta: -2.68, 95 %-KI: -5.08 bis -0.277, p=0.042)  Sicherheitsverhalten: Statistisch signifikanter Vorteil durch die Intervention (Beta: 0.05, 95 %-KI: 0.003 bis 0.097, p=0.037)  Gewaltexposition:  Psychologische Gewalt: Kein statistisch signifikanter Gruppenunterschied (x2: 2.20, p=0.333)  Körperliche Gewalt: Kein statistisch signifikanter Gruppenunterschied (x2: 0.75, p=0.687)  Sexuelle Gewalt: Kein statistisch signifikanter Gruppenunterschied (x2: 1.07, p=0.585)  Gewalt gegen Frauen (engl. "battering experience"): Kein statistisch signifikanter Gruppenunterschied (x2: 0.41, p=0.813)  Sekundäre Zielkriterien:  Depression: Kein statistisch signifikanter Gruppenunterschied (x2: 1.82, p=0.403)  Posttraumatische Belastungsstörung: Kein statistisch signifikanter Gruppenunterschied (x2: 0.58, p=0.750) |
