# Supplementary material for: Data Extractions Using a Large Language Model (Elicit) and Human Reviewers in Randomized Controlled Trials: A Systematic Comparison
Source: Cochrane Evid Synth Methods. 2025 Jun 8;3(4):e70033. doi: 10.1002/cesm.70033 (PMC12462964; doi:10.1002/cesm.70033)
Supplement: Supplementary file 3 — Appendix 3 Description of the commands by Elicit. [file CESM-3-e70033-s001.docx]

Appendix 3

Table 3: Description from the columnes from Elicit

| Columne | Command | Changes |
| --- | --- | --- |
| Study objectives | Be concise and use bullet points if there are several goals to the study. Only mention objectives that are stated as such by the authors; don't extrapolate | none |
| Sample characteristics | Characteristics include demographic information (age, sex, race, ethnicity, national origin, sexual orientation, religion, etc.), health status (healthy, cancer patients, obesity, etc.), and other relevant characteristics (e.g. occupation, socioeconomic status, etc.). Describe the effective Sample after Randomization. How many persons are recruited? Give the N. how many are in the intervention and control group. Use bullet points and aim to give one characteristic per bullet point. Example:  '- a total of 40 adult men and women aged 18-57, mean Age 30  - non-smokers  - majority white (68%)  - have a history of mental illness'  - 20 in the intervention group and 20 in the control group | We added:   - Effective Sample after Randomization - How many persons are recruited - Give the N - How many are in the intervention and control group - Example: a total of 40 adult men and women aged 18-57, mean Age 30 - 20 in the intervention group and 20 in the control group |
| Participant count | If randomization was done, give the number of participants randomized at the baseline or first phase of the study, not necessarily the number who were actually treated or analyzed. If not, give the number of participants. If available, state the number per each trial arm in the study (e.g. placebo vs. intervention), together with the arm names, using bullet points, e.g. 'Total: 446  - Placebo: 147  - Advil: 149  - Tylenol: 150', and ensure that they add up to the total. If the participants are not people, state the units (e.g. households, animals, etc.). | none |
| Study design | List all characteristics of study design, such as whether it was randomized, double-blind, controlled, placebo-controlled, non-controlled, multi-site, retrospective, stratified, crossover design, parallel design, an observational study, a meta-analysis, a systematic review, etc. Be comprehensive. If the study design is not mentioned, leave the answer blank | none |
| Intervention | List all interventions that at least some participants received and list the controls or placebos. If only some participants received a certain intervention, note that. Note the frequency, duration, and amount or dose of the intervention. Be as precise as possible: describe, how the intervention is used, if duration, frequency or dose are mentioned, they need to be in the answer. If there are multiple components to the intervention (e.g. a drug plus therapy), state all of them. Examples:  1. $199.92 USD per month for 23 days (short-term transfer group) or for 115 days (long-term transfer group).  2. 200mg ibuprofen and 200mg tylenol daily for 13 days.  3. the device is attached 5 cm from the finger | We added:   - And list the controls or placebos - How the intervention is used - The device is attached 5 cm from the finger |
| Outcome measured | There may be multiple primary outcomes or endpoints. If so, include all of them, as long as they are identified as main or primary outcomes or endpoints. Do not include secondary outcomes or endpoints. Include units if possible. For instance, if a study investigated the effects of caffeine on heart rate at rest, measured in bpm, and also measured as a secondary outcome the effect on anxiety, the answer is 'heart rate at rest (bpm)'. If the study investigated the effects of caffeine on heart rate, anxiety, and blood pressure, all of which seem to be equally important, then the answer is 'heart rate, anxiety, and blood pressure'. Be as precise as possible. | none |
| Intervention effect | Give all quantitative effects from the intervention(s) in the study, Be precise and exhaustive. Use numbers and bullet points. For all outcomes measured in the study, show the quantitative effects of each intervention and placebo/controls. Also give information on statistical significance if available (e.g. p values, or verbally saying 'significant'/'non-significant'). For example, if a study investigated the effects of vitamin E (intervention 1) and pioglitazone (intervention 2) on improvement in nonalcoholic steatohepatitis (outcome 1) and change in ALT levels (outcome 2), the answer might look like:  'improvement in nonalcoholic steatohepatitis:  - placebo: 19%  - Vitamin E therapy: 43% (p = 0.001 vs. placebo)  - pioglitazone: 34% (p = 0.04 vs. placebo)  Change in ALT levels:  - Placebo: -4 IU/L  - Vitamin E: -28 IU/L (p<0.001 vs placebo)  - Pioglitazone: -29 IU/L (p<0.001 vs placebo)' Remember to include all outcomes and results in your final answer and to answer in bullet points | none |
